# Helicase Domain of West Nile Virus NS3 Protein Plays a Role in Inhibition of Type I Interferon Signalling

**DOI:** 10.3390/v9110326

**Published:** 2017-11-02

**Authors:** Yin Xiang Setoh, Parthiban Periasamy, Nias Yong Gao Peng, Alberto A. Amarilla, Andrii Slonchak, Alexander A. Khromykh

**Affiliations:** Australian Infectious Diseases Research Centre, School of Chemistry and Molecular Biosciences, The University of Queensland, St. Lucia, QLD 4072, Australia; y.setoh@uq.edu.au (Y.X.S.); parthiban.periasamy@uq.edu.au (P.P.); y.g.peng@uq.net.au (N.Y.G.P.); a.amarillaortiz@uq.edu.au (A.A.A.); a.slonchak@uq.edu.au (A.S.)

**Keywords:** flavivirus, West Nile virus, NS3, helicase, interferon

## Abstract

West Nile virus (WNV) is a neurotropic flavivirus that can cause encephalitis in mammalian and avian hosts. In America, the virulent WNV strain (NY99) is causing yearly outbreaks of encephalitis in humans and horses, while in Australia the less virulent Kunjin strain of WNV strain has not been associated with significant disease outbreaks until a recent 2011 large outbreak in horses (but not in humans) caused by NSW2011 strain. Using chimeric viruses between NY99 and NSW2011 strains we previously identified a role for the non-structural proteins of NY99 strain and especially the NS3 protein, in enhanced virus replication in type I interferon response-competent cells and increased virulence in mice. To further define the role of NY99 NS3 protein in inhibition of type I interferon response, we have generated and characterised additional chimeric viruses containing the protease or the helicase domains of NY99 NS3 on the background of the NSW2011 strain. The results identified the role for the helicase but not the protease domain of NS3 protein in the inhibition of type I interferon signalling and showed that helicase domain of the more virulent NY99 strain performs this function more efficiently than helicase domain of the less virulent NSW2011 strain. Further analysis with individual amino acid mutants identified two amino acid residues in the helicase domain primarily responsible for this difference. Using chimeric replicons, we also showed that the inhibition of type I interferon (IFN) signalling was independent of other known functions of NS3 in RNA replication and assembly of virus particles.

## 1. Introduction

West Nile virus (WNV) causes West Nile encephalitis, the leading cause of arboviral encephalitis in the Americas. The North American strain (NY99) caused a large outbreak in 1999 in New York City and have since become endemic with annual seasonal outbreaks. To date, WNV have caused 46,289 reported cases with 2018 deaths in the United States of America (https://www.cdc.gov/westnile/statsmaps/index.html, accessed 14 September 2017). In Australia, Kunjin strain of WNV has been endemic since it was first discovered in 1960 but it has not caused significant outbreaks until the first large outbreak of equine encephalitis occurred in 2011 which was associated with the NSW2011 strain [1]. No human cases were reported and our initial characterisation of NSW2011 isolate revealed an intermediate virulence phenotype between the NY99 strain and the prototype MRM61C Kunjin strain in mice [1].

We then showed using chimeric viruses between NY99 and NSW2011 strains, that the non-structural region of NY99 was important for enhanced replication, inhibition of type I interferon (IFN) response and virulence [2]. Moreover, using chimeric viruses with replaced individual non-structural (NS) genes, we further defined that NY99 NS3 protein contributed to the most significant enhancement in virus replication and inhibition of IFN response [2].

The flavivirus genome is an approximately 11 kb positive-sensed RNA containing a 5′ cap (m7G5′ppp5′A) but lacking a 3′ poly(A) tail [3]. The genome encodes a polyprotein which is inserted into the membrane of the endoplasmic reticulum and post-translationally processed to yield three structural proteins (C, M and E) [4] and seven NS proteins (NS1, NS2A, NS2B, NS3, NS4A, NS4B and NS5). Host proteases process the polyprotein at sites in the endoplasmic reticulum lumen and a viral protease cleaves at specific sites in the cytoplasmic side of the endoplasmic reticulum membrane. Viral protease is encoded in the N-terminal domain of NS3 protein and is co-factored by the preceding NS2B protein. The C-terminal domain of NS3 encodes a viral RNA helicase that is classified within the superfamily 2 helicases [5]. This domain also encodes 5′-triphosphatase (NTPase) activity and 5′terminal RNA triphosphatase (RTPase) activity [6]. The helicase domain of NS3 works closely with the RNA-dependent RNA polymerase and capping activities of NS5 protein to replicate, unwind and cap viral RNA. In addition to its function in RNA replication, NS3 has also been shown to participate in the assembly of virus particles [7,8] although the exact mechanism of how NS3 performs this function remains unclear.

Apart from the known functions of NS3 in RNA replication and virus assembly, recent literature has indicated a role of flavivirus NS3 in interactions with the host innate immune system and in virus virulence. Dengue (DENV) NS2B/NS3 protease has been shown to be involved in inhibition of type I IFN production via specific cleavage of human stimulator of the interferon gene (STING) [9]. A set of paired mutations in envelope protein (E-402) and in the helicase domain of the NS3 protein (NS3-209/NS3-480) of DENV were found to have a synergistic effect in enhancing viral fitness in human and mosquito derived cell lines and produced a highly neurovirulent phenotype in mice [10]. DENV carrying single point mutations in the helicase domain of NS3 (L435S or L480S) replicated more efficiently and produced more viral progeny in infected monocyte-derived dendritic cells compared to the parental virus. This correlated with enhanced ATPase activity and modulation of the type I IFN response [11]. In WNV, a single amino acid substitution in helicase domain of NS3, T249P, has been shown to increase WNV viremia and associated mortality in crows but did not increase virus virulence in mice [12,13].

In this study, we expanded on our previous findings and identified the novel function for the helicase domain of WNV NS3 protein in inhibition of type I IFN signalling. We also showed that helicase of more virulent NY99 strain performed this function more efficiently than helicase of less virulent NSW2011 strain and identified two amino acid residues primarily responsible for this difference. Using chimeric replicons, we further showed that this new function of helicase domain was not linked to the previously known functions of NS3 in RNA replication and virus assembly.

## 2. Materials and Methods

### 2.1. Cells

Wild-type (wt) mouse embryonic fibroblasts (MEF), IRF-3^−/−^ × IRF-7^−/−^ MEF and IFNAR^−/−^ MEF were incubated in DMEM media (Invitrogen, Carlsbad, CA, USA) supplemented with 5% Foetal Bovine Serum (FBS), 10 U/mL penicillin and 10 μg/mL streptomycin. HEK293 cells were incubated in DMEM supplemented with 10% FBS, 10 U/mL penicillin, 10 μg/mL streptomycin and 1 mM sodium pyruvate.

### 2.2. Generation of Parental and Chimeric Viruses by Circular Polymerase Extension Reaction (CPER)

Table 1 details the list of primers used for generating NY99 and NSW2011 cDNA libraries. cDNA fragments for NY99 isolate 4132 were prepared by PCR amplification from the full-length cDNA template ligated from two plasmids [14]. cDNA fragments for NSW2011 were prepared by RT-PCR of viral RNA purified from NSW2011 virus isolated originally from the brain of an infected horse and then passaged once in C6/36 cells [1]. After RT-PCR amplification, the cDNA fragments were purified by gel extraction using the Monarch DNA Gel Extraction Kit (NEB, Ipswich, MA, USA), quantified using the Nanodrop 1000 (ThermoFisher Scientific, Waltham, MA, USA) and stored at −20 °C. For each CPER assembly, 0.06 pmol of each viral cDNA fragment was added to the PCR reaction using the PrimeSTAR GXL DNA Polymerase kit (Clontech, Mountain View, CA, USA) in a 50 μL reaction volume. Thermal cycling was carried out at 98 °C for 2 min (1 cycle), 98 °C for 10 s, 55 °C for 15 s, 68 °C for 12 min (20 cycles). The CPER reaction was transfected into HEK293 cells seeded in a 6 well plate using Lipofectamine LTX Plus reagent (Invitrogen). After 4–5 days of incubation, viruses in culture fluid were harvested (“passage 0” stock). Passage 1 viruses used in all experiments were produced in Vero cells by infecting T75 flasks with 200 μL of passage 0 stocks and harvesting culture fluids at 3–5 days post-infection. Virus titres were determined by plaque assay on baby hamster kidney (BHK) cells.

### 2.3. Plaque Assay

Plaque assays were performed as previously described [2].

### 2.4. Growth Kinetics

Wt or IFNAR^−/−^ MEF were infected at multiplicity of infection (MOI) = 0.1 and secreted viruses in the culture supernatant were harvested at 0, 24 and 48 h post-infection (hpi). Viral titres were determined by plaque assay on BHK cells. Five independent experiments were conducted.

### 2.5. Construction of Replicons

Fragments used for the construction of replicons include the C20DXGFPrep backbone—which contains the vector backbone including the SP6 promoter, 5′UTR, first 20 codons of Capsid and the green fluorescent protein (GFP) sequence—a synthetic DNA fragment containing the nanoLuciferase sequence (Promega, Madison, WI, USA) with complementary ends to GFP sequence and NS1 signal sequence (end of E). The remaining fragments were amplified using primers designed similarly to the CPER for generating NSW2011 and chimeric WN viruses (Table 1) [2,15]. A total of 0.1 pmols of each DNA fragment were mixed into an equal volume of Gibson Assembly Master Mix (NEB) and incubated at 50 °C for 2 h, before electroporation into DH5α cells for recovery of assembled replicons.

### 2.6. RNA Replication and Virus-Like Particle (VLP) Production Assays

To assess RNA replication efficiencies, replicon RNAs were first transcribed in vitro from *XhoI*-linearised template DNA using SP6 RNA polymerase (Roche, Basel, Switzerland) and capping analogue, m7G(5′)ppp(5′)G (NEB) and then electroporated into BHK cells as described previously [16]. At 0, 24 and 48 h post transfection, cell lysates were harvested in Glo-Lysis buffer (Promega) and then luciferase activity was quantified using the Nano-Glo Luciferase Assay System (Promega). To assess the production of virus-like particles (VLPs), replicon RNAs were in vitro transcribed as above and then electroporated into a tetracycline-inducible packaging BHK cell line, tetKUNCprME that produces Kunjin (KUN) structural proteins C, prM and E [17]. tetKUNCprME cells were cultured in the presence of puromycin (10 μg/mL), G418 (0.5 mg/mL) and doxycycline (0.5 μg/mL). Doxycycline was removed from the medium immediately after electroporation of replicon RNA to induce expression of CprME and enable VLP production. Culture supernatant were collected at 24, 48 and 72 h post electroporation and VLP titres were determined using VLP titration assay.

### 2.7. VLP Titration Assay

Vero cells were seeded at a density of 1 × 10^6^ cells per 96-well plate one day before the assay. Culture supernatant containing VLPs were pre-diluted in a separate 96-well plate in 10-fold serial dilutions. Media from the Vero cell plate were removed, 100 μL of pre-diluted samples were added and cells were incubated at 37 °C in a CO_2_ incubator for 72 h. Media was then removed and 80% ice-cold acetone was added into the wells and incubated at −20 °C for 30 min. Plates were then left to completely dry (for approximately 6 h), before probing with anti-GFP rabbit antibody (Cat# A6455, Life Technologies, Carlsbad, CA, USA) and anti-rabbit IgG AlexaFluor 680 (Life Technologies). Cells were washed once with PBS supplemented with 0.2% Tween-20 (PBS/T) between each antibody step. After the final wash, remaining PBS/T were removed and plates were allowed to dry and then scanned on the Odyssey scanner (LI-COR, Lincoln, NE, USA) at a resolution of 21 μm, medium quality setting and a focal length of 3 mm.

### 2.8. Flow Cytometry

IRF-3^−/−^ × IRF-7^−/−^ MEF were seeded onto 6 well plates at a density of 5 × 10^5^ cells per well and infected the next day with viruses at MOI 1. At 48 hpi, cells were treated with 10,000 IU of IFN-α for 30 min and then trypsinised, collected in 1 mL PBS and pelleted by centrifugation. The cell pellet was resuspended in 200 μL PBS and fixed by adding 800 μL of 4% formaldehyde and incubated at 37 °C for 10 min. The fixed cells were pelleted by centrifugation and permeabilized by resuspending in 200 μL of ice-cold 90% methanol and incubated on ice for 30 min. The cells were either stored in methanol at −20 °C, or resuspended in PBS for immunostaining. Briefly, the fixed and permeabilized cells were stained using the anti-NS1 4G4 mAb [18] and Phospho-Stat1 (Tyr701) (58D6) rabbit mAb (Cell Signaling Technology, Danvers, MA, USA) and then labelled with secondary goat anti-mouse IgG-Alexa Fluor 488 antibody and goat anti-rabbit IgG-Alexa Fluor 647 (Invitrogen). Cells were washed twice with PBS/T between each antibody step. Stained cells were analysed using the BD Accuri C6 Flow Cytometer (BD biosciences, Franklin Lakes, NJ, USA). Four independent experiments were performed.

### 2.9. Virulence in Mice

All animal procedures were approved by The University of Queensland Animal Ethics committee in accordance with the guidelines set by the National Health and Medical Research Council, Australia. The Animal Ethics Approval Certificate number is AIBN/327/15/NHMRC, approved on 15 September 2015. Groups of ten 4-week-old female Swiss outbred mice (Animal Resources Centre, Murdoch, Western Australia, Australia) were infected intraperitoneally (i.p.) with 1000 plaque forming units (pfu)/mouse of each virus based on viral titres determined by plaque assay of Vero cells. After infection, animals were monitored for the signs of disease daily for a total of 21 days and scored for the observed symptoms as follows: score 0—normal feeding and appearance; score 1—slightly ruffled fur and/or general loss of condition; score 2—increases in above behaviour/appearance, breathing changes, twitching, anti-social behaviour, score 3—severely hunched posture or partial paralysis (e.g., some immobility, unsteady gait, flaccid hind legs, sever twitching/fitting) or full paralysis. Once the score reached 3 the animal was immediately sacrificed.

### 2.10. Statistical Analyses

All data were analysed by two-way ANOVA with multiple comparison (GraphPad version 7.03, La Jolla, CA, USA), except the survival curves which were analysed using the Log-rank (Mantel-Cox test) (GraphPad).

## 3. Results

### 3.1. Helicase Domain of NY99 NS3 Protein Is Responsible for Enhanced Virus Replication in Type I IFN Response-Competent Mouse Cells

**Figure 1:**
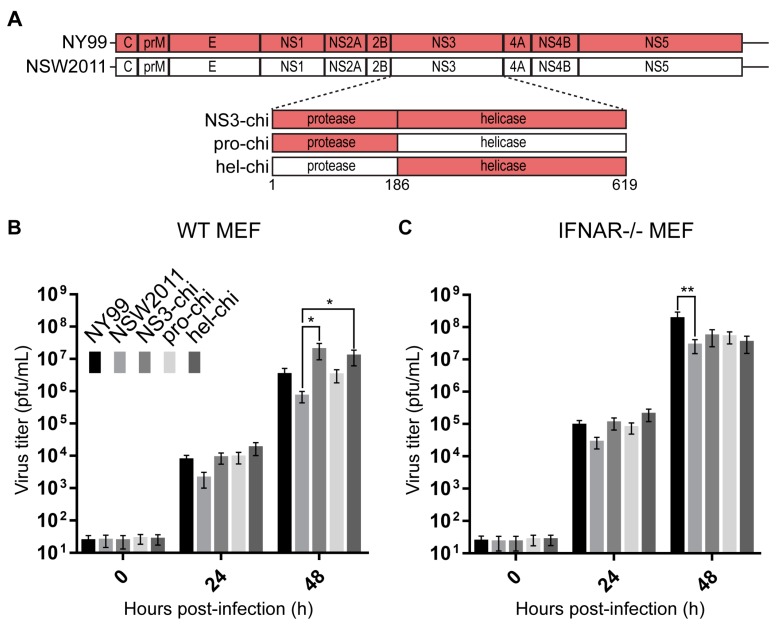
Construction and characterisation of NSW2011, NY99 and chimeric viruses. (**A**) Schematic of NSW2011, NY99 and NS3 (NS3-chi), protease (pro-chi) and helicase (hel-chi) chimeric viruses. Growth kinetics of parental and chimeric viruses in (**B**) WT MEF and (**C**) IFNAR^−/−^ MEF infected at an MOI of 0.1. Culture supernatant were harvested at the indicated time points and titrated by plaque assay. Five independent experiments conducted. Error bars indicate mean ± standard error and statistical analysis performed using two-way ANOVA with multiple comparison. * *p* ≤ 0.05, ** *p* ≤ 0.01.

We previously demonstrated that the NSW2011/NY99-NS3 chimeric virus exhibited enhanced virus replication in the wild type mouse embryonic fibroblasts (WT MEF) and increased virulence in mice [2]. To further define which functional domain of NS3 was responsible for this phenotype, NSW2011/NY99-protease (pro-chi) and NSW2011/NY99-helicase (hel-chi) chimeric viruses, encoding either protease or helicase domains of NY99 NS3 on the background of NSW2011 genome, were generated using our previously described CPER method (Figure 1A) [2,15,19,20].

To assess the potential role of helicase and protease domains of NS3 in inhibition of type I IFN response, multi-step virus growth kinetics were performed using WT MEF as well as IFN-α/β receptor knockout (IFNAR^−/−^) MEF, at an MOI of 0.1. At 48 h post-infection (hpi), NS3-chi grew to significantly higher titres than NSW2011 in WT MEF (Figure 1B), as expected from our previous results [2]. Of the two chimeric viruses with individual domains of NS3 replaced, only hel-chi but not pro-chi grew to significantly higher titre in WT MEF than NSW2011 at 48 hpi (Figure 1B). This enhancement in virus replication by the helicase domain was not observed in IFNAR^−/−^ MEF (Figure 1C), suggesting that the helicase domain of NY99 NS3 may be responsible for more efficient inhibition of type I IFN response.

### 3.2. RNA Replication and Virus Assembly Is Not Enhanced by the Helicase Domain of NY99 NS3 Protein

**Figure 2:**
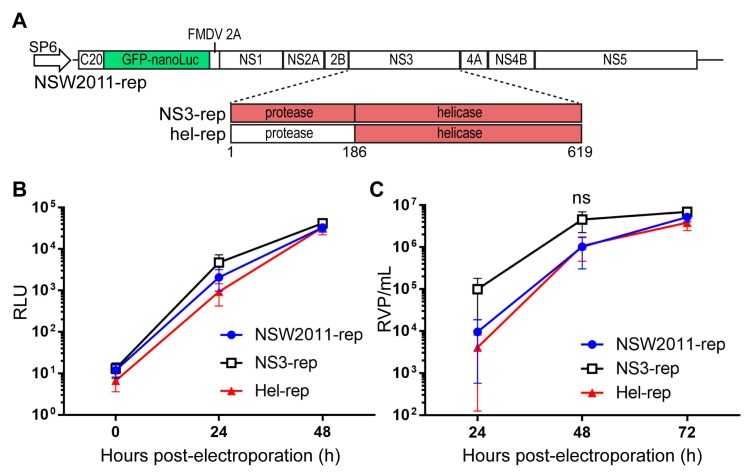
Analysis of RNA replication and VLP production efficiencies of NSW2011 and chimeric replicons. (**A**) Schematic of NSW2011, NSW2011/NY99-NS3 (NS3-rep) and NSW2011/NY99-helicase (hel-rep) chimeric replicons; (**B**) BHK cells were electroporated with the respective replicon RNAs and nano-luc activity in cell lysates was determined at indicated time points. Error bars indicate mean ± standard error. Four independent experiments were performed; (**C**) tetKUNCprME BHK packaging cells were electroporated with the respective replicon RNAs and culture supernatant harvested at the indicated time points after electroporation. VLP titres were determine using a VLP assay on Vero cells. Two independent experiments conducted. Error bars indicate mean ± standard error and statistical analysis performed using two-way ANOVA with multiple comparison. ns = not significant.

To determine if the enhanced replication of hel-chi in WT MEF was also aided by the other known functions of the NS3-helicase in RNA replication and virus assembly, we constructed the NSW2011 replicon (NSW2011-rep) expressing the GFP-nano-Luciferase fusion protein, as well as the NSW2011/NY99-NS3 chimeric replicon (NS3-rep) and the NSW2011/NY99-helicase chimeric replicon (hel-rep) (Figure 2A). RNA replication efficiencies were analysed by electroporating in vitro transcribed replicon RNAs into BHK-21 cells and determining luciferase activity, as a measure of RNA replication efficiency, at 0, 24 and 48 h post-electroporation (Figure 2B). No significant differences in RNA replication efficiencies were observed between all three replicons, indicating that RNA replication efficiency was not influenced by the NY99 NS3 protein or its helicase domain. We also wanted to determine if the enhanced virus replication of NS3-chi and hel-chi in WT MEFs were aided by the function of NS3 in RNA packaging/virus assembly. To this end, we performed replicon RNA packaging assay by electroporating BHK packaging cell line [17] with the in vitro transcribed replicon RNAs and determined titres of secreted VLPs at 24, 48 and 72 h post-electroporation (Figure 2C). Similarly, no statistically significant differences in the titres of secreted VLPs were observed, indicating that the observed enhanced replication of NS3-chi and hel-chi viruses in WT MEF was not linked to the functions of NS3 in RNA replication and RNA packaging/virus assembly.

### 3.3. NS3-Helicase Domain of NY99 Inhibits Phosphorylation of STAT1

**Figure 3:**
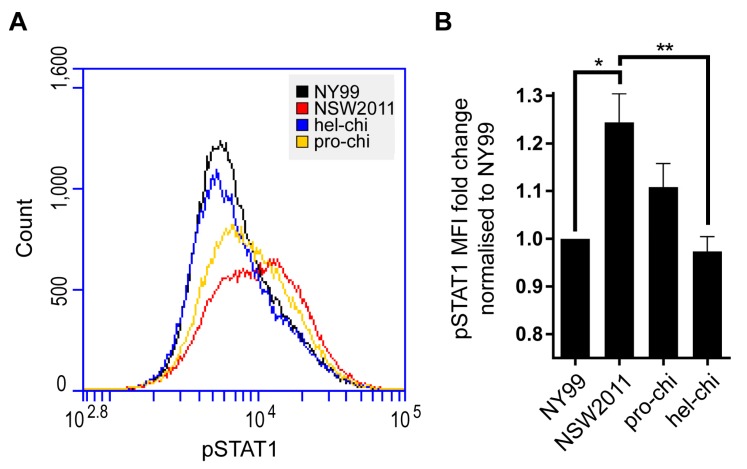
Analysis of STAT1 phosphorylation in cells infected with NSW2011, NY99 and chimeric viruses. (**A**) IRF-3^−/−^ × IRF-7^−/−^ MEF were infected with NY99, NSW2011, pro-chi and hel-chi at MOI 1. At 48 h post-infection, cells were treated with 10,000 International Units (IU) per mL of IFN-α and then fixed and stained for pSTAT1 and WNV NS1. Cells were first gated for infected cells that express NS1 and then pSTAT1 levels were determined in gated infected cell population; (**B**) Four independent experiments were performed and pSTAT1 mean fluorescent intensity (MFI) fold change was calculated and normalised to NY99. Error bars indicate mean ± standard error. Statistical analysis performed using two-way ANOVA with multiple comparison. * *p* ≤ 0.05, ** *p* ≤ 0.01.

As another WNV protein NS5, localised in the cytoplasm, was previously shown to modulate the type I IFN response by inhibiting STAT1 phosphorylation [21], we were interested to determine if the cytoplasmically localised NS3 also employed the same strategy. We choose to use IRF-3^−/−^ × IRF-7^−/−^ MEF that should not produce appreciable levels of endogenous type IFN upon WNV infection [22,23] and thus should not compromise downstream readouts of pSTAT1 expression in our assays. We infected IRF-3^−/−^ × IRF-7^−/−^ MEF with NY99, NSW2011, pro-chi, hel-chi viruses for 48 h, treated with 10,000 IU of IFN-α for 30 min and then fixed and stained with antibodies to viral NS1 protein and to pSTAT1 for analysis by flow cytometry. Infected cells were first gated based on expression of NS1 (infected) and then further analysed for expression of pSTAT1. NY99-infected and hel-chi-infected cells showed significantly lower pSTAT1 expression compared to NSW2011-infected cells, indicating more efficient inhibition of STAT1 phosphorylation, while pSTAT1 expression levels in NSW2011-infected and pro-chi-infected cells were not significantly different (Figure 3A,B).

### 3.4. Proline at Position 249 and Phenylalanine at Position 486 in the Helicase Domain of NY99 NS3 Are Primarily Responsible for the Inhibition of STAT1 Phosphorylation

**Figure 4:**
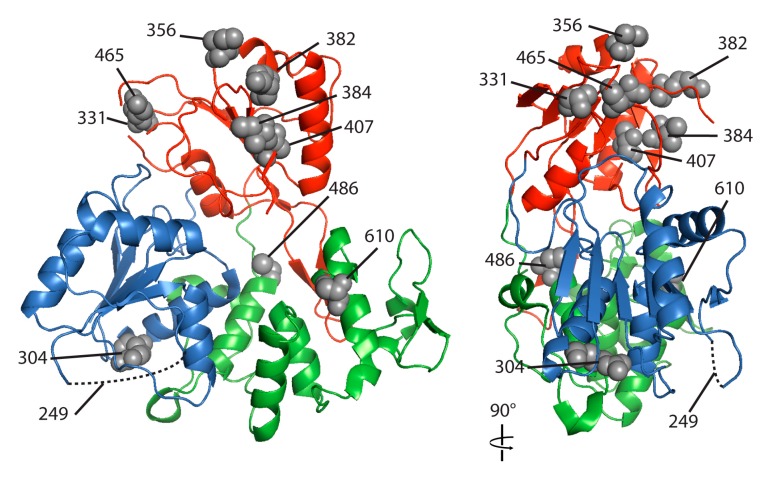
Crystal structure of WNV (Kunjin) helicase. Crystal structure of WNV Kunjin helicase (PDB: 2QEQ) [6] showing domain I (blue), domain II (red) and domain III (green). Grey spheres represent the amino acids different in the NSW2011 and the NY99 viruses, indicated with their corresponding residue numbers. Residue 249 is located within an unresolved solvent exposed loop in domain I (indicated by dotted line).

Out of the ten amino acid substitutions in helicase domain between NSW2011 and NY99 (Table 2), six are within domain II (DII) of helicase, while DI and DIII have two substitutions each. Based on the crystal structure of Kunjin virus (KUNV) helicase (PDB: 2QEQ), the 6 substitutions in DII are clustered towards the apical surface of DII (Figure 4). In DI, the substitution A249P is found in the solvent-exposed flexible loop and R304K is found in the α-helix 4 of DI, the latter is predicted not to affect the α-helix structure. DI and DII likely originated from gene duplication [24] and DIII is considered a unique structure and does not match with any known structures [25]. DIII is linked and stabilised by a protruding β-hairpin from DII which interacts with the short β-strand residues 596-598 of DIII. One of the substitutions (C486F) sits on the N-terminal of the first alpha-helix of DIII, which is positioned directly facing the DII protruding beta-hairpin. The second change in DIII is S610A (in DIII α-helix 9), which forms the bottom part of the ssRNA entry clef.

**Figure 5:**
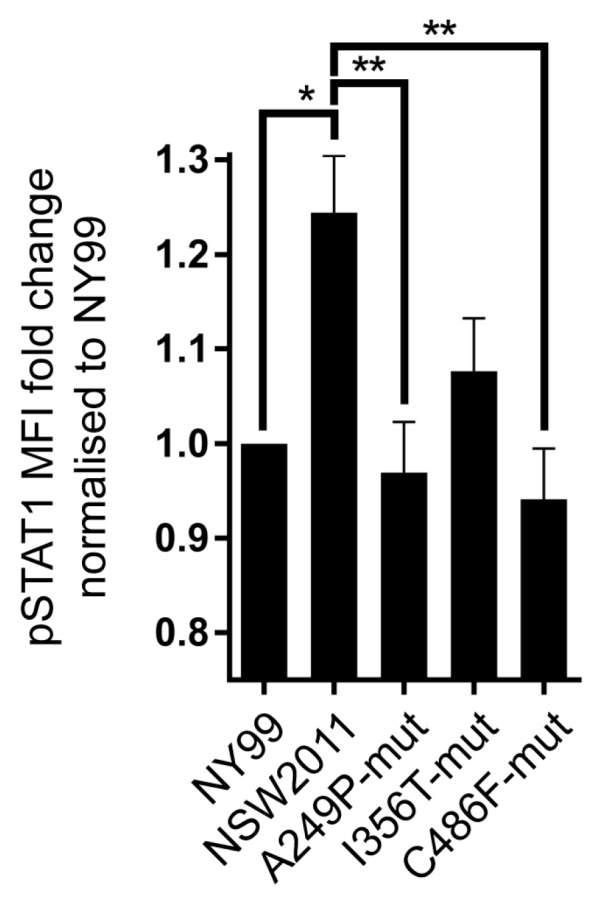
Analysis of STAT1 phosphorylation in cells infected with mutant NSW2011 viruses. IRF-3^−/−^ × IRF-7^−/−^ MEF were infected with NY99, NSW2011 and A249P, I356T and C486F mutant NSW2011 viruses at MOI 1. At 48 h post-infection, cells were treated with 10,000 IU of IFN-α for 30 min, fixed, stained for NS1 and pSTAT1 and analysed by flow cytometry. Four independent experiments were conducted and pSTAT1 fold change was calculated and normalised to NY99. Error bars indicate mean ± standard error. Statistical analysis performed using two-way ANOVA with multiple comparison. * *p* ≤ 0.05, ** *p* ≤ 0.01.

To assess the role of individual residues that are different between NY99 and NSW2011 helicase domains in the inhibition if type I IFN signalling, we elected to generate three individual amino acid mutant viruses that presented the most non-conservative changes. A249P-mut introduces proline instead of alanine that results in a turn in the protein structure, I356T-mut changes hydrophobic isoleucine to a neutral threonine residue and C486F-mut replaces highly reactive cysteine to less reactive phenylalanine. The mutations were introduced into the NSW2011 genome and the resultant viruses were examined for their ability to inhibit STAT1 phosphorylation in infected IRF-3^−/−^ × IRF-7^−/−^ MEF in response to the treatment with exogenous IFN, as above. The infection with A249P-mut and C486F-mut viruses but not with I356T-mut virus, resulted in significantly more efficient inhibition of STAT1 phosphorylation in response to IFN treatment, as compared to the NSW2011 infection (Figure 5).

### 3.5. Helicase Domain of NY99 NS3 Does Not Significantly Enhance Virulence in Mice

**Figure 6:**
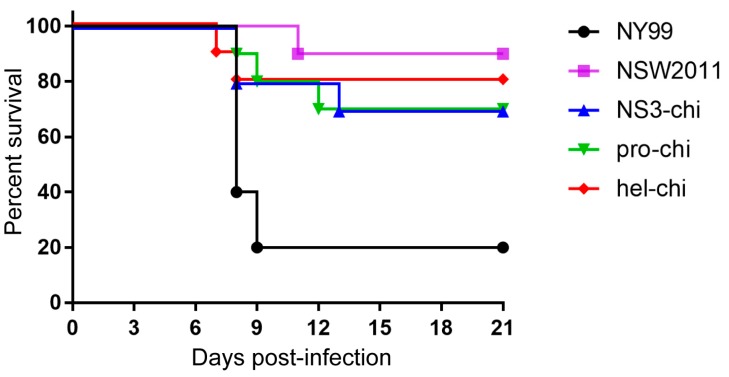
Virulence of NSW2011, NY99 and chimeric viruses in mice. Four weeks old female Swiss outbred CD1 mice (10 per group) were infected with 1000 pfu of NY99, NSW2011, NS3-chi, pro-chi and hel-chi viruses via the intraperitoneal (i.p.) route. Animals were monitored daily for 21 days post infection and were sacrificed when signs of encephalitis were observed.

To determine if NY99 helicase and its associated function in inhibition of IFN signalling contributed to virulence in vivo, groups of 10 four-week old CD1 mice were infected intraperitoneally with 1000 pfu of NY99, NSW2011, NS3-chi, pro-chi, or hel-chi viruses and monitored for 21 days. NY99 was significantly more virulent than NSW2011 (*p* = 0.0008, Log-rank Mantel-Cox test), as expected (Figure 6). Although a trend for increased virulence for chimeric viruses compared to NSW2011 has emerged, the differences were not statistically significant (Figure 6). The results suggest that other NY99 viral proteins in addition to NS3 are required for higher virulence in mice.

## 4. Discussion

WNV is one of the most geographically widespread arboviruses in the world and the leading cause of arboviral encephalitis [26]. In North America, annual recurrence of WNV outbreaks have become a regular feature since its introduction in 1999. Kunjin virus has traditionally been associated with mild and rare disease in humans and horses in Australia [27], however, following extensive flooding across Eastern Australia in 2011, an unprecedented outbreak of equine encephalitis occurred, leading to the isolation of the first virulent Kunjin strain to cause a major outbreak (NSW2011), albeit confined to horses [28]. We have initially characterised the NSW2011 virus and showed that it presented an intermediate virulence phenotype in mice compared to the prototype MRM61C Kunjin and NY99 strains [1] and further defined by using chimeric viruses that replacing the region encoding non-structural proteins in NSW2011 with those of NY99 strain resulted in enhanced virulence in mice [2]. Among the individual non-structural proteins, the chimeric NSW2011 virus containing NY99 NS3 protein (NSW2011/NY99-NS3) was found to be the most efficient in replication in type I IFN response-competent cells [2], suggesting a role for NS3 in inhibition of IFN response. In this study, we aimed to determine if the protease or helicase domain of NS3 were responsible for the enhanced replication and inhibition of type I IFN response and identify steps in type I IFN response targeted for inhibition.

Reaffirming our previous findings, the NSW2011/NY99-NS3 chimeric virus displayed enhanced replication in IFN response-competent WT MEFs and this was attributed to the helicase domain of NY99 NS3, as demonstrated by the enhanced replication of the hel-chi virus. Thus, we show here for the first time that WNV NS3 helicase plays a role in inhibition of the type I IFN response. Point mutations in DENV NS3 helicase were previously reported to enhance virus ability to circumvent type I IFN response but this was associated with increased viral RNA replication caused by enhanced ATPase activity [11]. In our study, RNA replication of hel-chi replicon was not more efficient than that of NSW2011 replicon in type I IFN response-deficient BHK cells and also the replication of hel-chi virus was not more efficient than that of NSW2011 virus in type I IFN response-deficient IFNAR^−/−^ MEF, thus demonstrating that the function of NY99 NS3 helicase in inhibition of IFN response is not directly linked to its function in RNA replication. The other known function of NS3 in virus assembly was also not responsible for enhanced replication of hel-chi in WT MEF, as packaging efficiencies of both, NS3-rep and hel-rep, replicon RNAs into VLPs were not significantly higher than that of parental NSW2011-rep RNA. Thus, our results demonstrate that the function of NY99 helicase domain of NS3 in inhibition of type I IFN response is not linked to its other functions in RNA replication and packaging/virus assembly.

Our experiments on defining the role of NS3 and its helicase domain in inhibition of type IFN signalling utilised IRF-3^−/−^ × IRF-7^−/−^ MEF, which do not produce appreciable levels of endogenous type I IFNs upon WNV infection [22,23]. The lack of endogenous type I IFN response to infection in these cells thus allows accurate quantification of the inhibition of type I IFN signalling induced by addition of exogenous IFNβ. The accurate quantification of STAT1 phosphorylation was further facilitated by analysing pSTAT1 levels only in infected cells via gating cell population for WNV NS1 expression. The results narrowed down the mechanism underlying the enhanced replication of the hel-chi virus in a type I IFN response-competent environment to potential interactions of helicase domain with the host factor(s) in the type I IFN signalling pathway acting between IFNAR1/IFNAR2 dimerization and phosphorylation of STAT1.

With the aim of further resolving the specific residues in helicase that were involved in inhibition of STAT1 phosphorylation, we selected the three most non-conserved amino acid changes between NSW2011 and NY99 helicase, A249P, I356T and C486F and generated corresponding mutant NSW2011 viruses. A249P and C486F mutant viruses but not I356T, showed enhanced inhibition of STAT1 phosphorylation, which was similar to that exhibited by the NY99 virus. It is not clear how these two separate amino acid changes are independently able to inhibit STAT1 phosphorylation. It is possible that these two mutations may individually affect the overall structure of the helicase domain that may be required for interactions with the host factors acting between interferon-alpha/beta receptor (IFNAR) dimerization and STAT1 phosphorylation. The T249P mutation in NS3, for example, was previously reported to be important for NY99 virulence in the avian model but had no effect on virulence in mice [12]. Replication of the 249P virus was shown to be less temperature sensitive at 44 °C (average avian temperature) compared to 37 °C, whereas the 249T virus was significantly more temperature sensitive at 44 °C [12,13], possibly explaining higher virulence of 249P virus in avian hosts. However, temperature sensitivity of some of the other viral mutants at this position (e.g., Asp or His) did not correlate with their virulence in avian hosts. No difference in helicase activity was observed between 249P and other recombinant helicases containing different amino acids at this position, while 249P recombinant helicase showed higher in vitro ATPase activity compared to 249A or 249T helicases [12,13]. How this increased in vitro ATPase activity and lower temperature sensitivity could contribute to higher virulence of 249P virus in avian hosts but not in mice, remains unclear. It is also not resolved how other residues at this position could contribute to differential virulence in avian and mammalian hosts. Our results show significant differences between 249P (NY99) and 249A (NSW2011) viruses as well as between 486F (NY99) and 486C (NSW2011) viruses in their abilities to inhibit type I IFN signalling in MEFs. Why incorporation of the helicase domain or even the entire NY99 NS3 protein into NSW2011 virus did not significantly increase virulence in mice remains unclear and suggests a role for a combination of NS3 and other viral proteins in higher virulence of NY99 virus in mammals. It is also possible that the amino acid changes in the helicase domain of NS3 identified in our study may have an effect on virulence in avian hosts which, unlike mammals, represent the main amplifying hosts in the WNV life cycle. This clearly warrants further investigation.

Overall, our study identifies WNV NS3 helicase as yet another viral protein that targets the host type I IFN signalling pathway and warrants further studies to determine the specific mechanism and interactions involved.

## Figures and Tables

**Table 1:** Primers used for the generation of CPER amplicons.

Amplicon Name	Primer Name	Primer Sequence (5′–3′)
UTRlinker	flaviUTRlinker_F	GTGGTGCGAGAACACAGGA
	flaviUTRlinker_R	CAGCTCACACAGGCGAACTACT
5′UTR	5′UTR_F	AGTAGTTCGCCTGTGTGAGCTG
	5′UTR_R	GGCCCTCCTGGTTTCTTAGAC
C-prM-E	CprME_F	GTCTAAGAAACCAGGAGGGCC
	CprME_R	GGAAAGAAAGAGCAGAACTCCTCCAAC
NS1-2A-2B	NS1_F	GTTGGAGGAGTTCTGCTCTTTCTTTCC
	NS2B_R	CTCCTCTCTTTGTGTATTGGAGAGTTATC
NS3-4A-4B	NS3_F	GATAACTCTCCAATACACAAAGAGAGGAG
	NS4B_R	CGTCCTTTTGCCCCACCTC
NS5-3′UTR	NS5_F	GAGGTGGGGCAAAAGGACG
	3′UTR_R	TCCTGTGTTCTCGCACCAC
NS1	NS1_F	GTTGGAGGAGTTCTGCTCTTTCTTTCC
	NS1_R	GGCCAAGAACACGACCAGAAG
NS2A	NS2A_F	CTTCTGGTCGTGTTCTTGGCC
	NS2A_R	CAGCTGTCATCACTTCAGTTGC
NS2B	NS2B_F	GCAACTGAAGTGATGACAGCTG
	NS2B_R	CTCCTCTCTTTGTGTATTGGAGAGTTATC
NS3	NS3_F	GATAACTCTCCAATACACAAAGAGAGGAG
	NS3_R	CCTGAGGCGAAGTCTTTGAA
NS4A	NS4A_F	TTCAAAGACTTCGCCTCAGG
	NS4A_R	CCCATCTCATTGGCTGCCAC
NS4B	NS4B_F	GTGGCAGCCAATGAGATGGG
	NS4B_R	CGTCCTTTTGCCCCACCTC
Helicase chimera	Hel_F	CGGATTCGAACCTGAGATGTTGAGG
	NS3_R	CCTGAGGCGAAGTCTTTGAA
Protease chimera	NS3_F	GATAACTCTCCAATACACAAAGAGAGGAG
	Pro_R	CCTCAACATCTCAGGTTCGAATCCG

**Table 2:** Amino acid substitutions in NS3 protein between KUNV, NSW2011 and NY99.

Domain	Amino Acid Position	Domain	KUNV	NSW2011	NY99
Protease	110		Q	Q	R
	175		V	V	I
Helicase	249	1	A	A	P
	304	1	R	R	K
	331	2	A	A	S
	356	2	I	I	T
	382	2	K	R	K
	384	2	I	I	V
	407	2	V	V	I
	465	2	N	S	N
	486	3	C	C	F
	610	3	S	S	A
